# Untargeted Metabolomic Analysis of Human Milk from Mothers of Preterm Infants

**DOI:** 10.3390/nu13103604

**Published:** 2021-10-14

**Authors:** Lila S. Nolan, Angela N. Lewis, Qingqing Gong, James J. Sollome, Olivia N. DeWitt, Robert D. Williams, Misty Good

**Affiliations:** 1 Department of Pediatrics, Washington University School of Medicine, St. Louis, MO 63110, USA; lilanolan@wustl.edu (L.S.N.); qgong@wustl.edu (Q.G.); dewitton@wustl.edu (O.N.D.); robert.d.williams@wustl.edu (R.D.W.); 2 Department of Pediatrics, Saint Louis University School of Medicine, St. Louis, MO 63104, USA; angela.lewis.1@health.slu.edu; 3 Metabolon, Inc., Morrisville, NC 27560, USA; jsollome@metabolon.com

**Keywords:** breast milk, necrotizing enterocolitis, metabolomics, metabolites, human milk, prematurity, newborn

## Abstract

The application of metabolomics in neonatology offers an approach to investigate the complex relationship between nutrition and infant health. Characterization of the metabolome of human milk enables an investigation into nutrients that affect the neonatal metabolism and identification of dietary interventions for infants at risk of diseases such as necrotizing enterocolitis (NEC). In this study, we aimed to identify differences in the metabolome of breast milk of 48 mothers with preterm infants with NEC and non-NEC healthy controls. A minimum significant difference was observed in the human milk metabolome between the mothers of infants with NEC and mothers of healthy control infants. However, significant differences in the metabolome related to fatty acid metabolism, oligosaccharides, amino sugars, amino acids, vitamins and oxidative stress-related metabolites were observed when comparing milk from mothers with control infants of ≤1.0 kg birth weight and >1.5 kg birth weight. Understanding the functional biological features of mothers’ milk that may modulate infant health is important in the future of tailored nutrition and care of the preterm newborn.

## 1. Introduction

Human breast milk metabolomics aims to identify the complete set of low molecular weight metabolites in maternal milk. This is a promising approach for metabolic fingerprinting and phenotyping of maternal milk to highlight variations in composition related to maternal health and diet and the resulting impact on the infant. Variations in the bioactive components of human milk have been shown to influence neonatal health outcomes, including growth, development, protection from disease development, neurodevelopment, and immunological development [1,2,3,4,5,6,7,8,9].

Few studies have explored the comprehensive metabolome of human breast milk. Proton (^1^H) nuclear magnetic resonance (NMR) spectroscopy and mass spectrometry (MS) are the most employed methods for metabolomic analysis of human milk. The first human milk metabolomic analysis was performed in 2012 as a potential screening tool for milk composition in the first month of lactation for mothers with preterm low birth weight infants using 27 human milk samples [10]. When compared to infant formula, significant differences were identified in the metabolic profile comparison, with preterm maternal milk containing higher concentrations of lactose and formula containing higher concentrations of maltose, oleic and linoleic acids [10].

The investigation into the benefits of human milk and neonatal disease prevention is an active field of study in neonatology. Metabolomic analyses of human milk have extensively explored human milk oligosaccharides (HMOs), the third most abundant component in milk after lactose and fat [11]. Other bioactive metabolites, including those derived from fatty acids, amino sugars, amino acids, microbial byproducts, vitamins and oxidative stress-related compounds, are multipurpose and have known protective roles against the pathogenesis of neonatal diseases such as necrotizing enterocolitis (NEC). In high-risk preterm infants, dysregulation of barrier integrity and immunity of the intestine contribute to the development of NEC, a gastrointestinal emergency that affects approximately 10% of infants with a birth weight <1500 g [12,13]. It has been shown that an early and exclusive human milk diet is associated with a lower risk of developing NEC in very low birth weight infants [14,15]. In this study, we aim to examine the global metabolomic profile of human milk in preterm infants who developed NEC versus age-matched control preterm infants. We hypothesized that metabolites of human milk from mothers of infants with NEC may be distinctly different from the metabolomic profile of milk from mothers of healthy preterm controls. Further, we examined the effect of birth weight on breast milk composition in non-NEC infants.

## 2. Materials and Methods

### 2.1. Subjects and Samples

Following consent, samples of breast milk from mothers of infants admitted to St. Louis Children’s Hospital neonatal intensive care unit (NICU) were obtained as part of a longitudinal cohort study with institutional IRB approval. After a sufficient volume of maternal breast milk could be expressed to meet an infant’s nutritional requirements, up to 3 mL of breast milk was collected for this study. Unfortified breast milk samples from 48 mothers with preterm infants (<37 weeks) included in this study were collected between Aug 2017 and Nov 2020, and were stored in 1 mL aliquots at −80 °C within 24 h of collection. Samples from mothers whose infants developed NEC were matched with a control sample obtained on a similar postnatal day of an infant of a similar corrected age. Additional samples from mothers of infants who did not develop NEC were combined with the corrected age-matched control samples for a comparison of a ≤1.0 kg and a >1.5 kg birth weight group. For sample processing, banked samples were thawed on ice and 250 µL of milk was aliquoted into sterile polypropylene tubes. Clinical information from the medical record was also obtained for study subjects, including maternal prenatal history, delivery history and NICU hospitalization course of the infant.

### 2.2. Sample Preparation

Samples were prepared by Metabolon, Inc. with the automated MicroLab STAR^®^ system from Hamilton Company using an established method [16,17]. Proteins were precipitated with methanol under vigorous shaking for two minutes (Glen Mills GenoGrinder 2000, Clifton, NJ, USA) followed by centrifugation to remove protein and to recover chemically diverse metabolites. The resulting extract was divided into five fractions: two for analysis by two separate reverse phase (RP)/Ultrahigh Performance Liquid Chromatography-Tandem Mass Spectroscopy (UPLC-MS/MS) methods with positive ion mode electrospray ionization (ESI), one for analysis by RP/UPLC-MS/MS with negative ion mode ESI, one for analysis by HILIC/UPLC-MS/MS with negative ion mode ESI, and one sample was reserved for backup. Samples were placed briefly on a TurboVap^®^ (Zymark, Hopkinton, MA, USA) to remove the organic solvent. The sample extracts were stored overnight under nitrogen before preparation for analysis.

### 2.3. QC

Several controls were analyzed in concert with the experimental samples: a pooled matrix sample generated by taking a small volume of each experimental sample (or alternatively, the use of a pool of well-characterized human plasma), which served as a technical replicate throughout the data set; extracted water samples served as process blanks; and a cocktail of QC standards that were carefully chosen not to interfere with the measurement of endogenous compounds were spiked into every analyzed sample, allowed instrument performance monitoring and aided chromatographic alignment. Experimental samples were randomized across the platform run with QC samples spaced evenly among the injections [17].

### 2.4. Ultrahigh Performance Liquid Chromatography-Tandem Mass Spectroscopy (UPLC-MS/MS)

All methods utilized an ACQUITY ultra-performance liquid chromatography (UPLC) (Waters, Milford, MA, USA), a Q-Exactive high resolution/accurate mass spectrometer interfaced with a heated electrospray ionization (HESI-II) source (Thermo Scientific, Waltham, MA, USA) and Orbitrap mass analyzer operated at 35,000 mass resolution [17]. The sample extract was dried and reconstituted in solvents compatible with each of the four methods. Each reconstitution solvent contained a series of standards at fixed concentrations to ensure injection and chromatographic consistency. One aliquot was analyzed using acidic positive ion conditions, chromatographically optimized for more hydrophilic compounds. The extract was gradient eluted from a C18 column (Waters UPLC BEH C18—2.1 × 100 mm, 1.7 µm) using water and methanol, containing 0.05% perfluoropentanoic acid (PFPA) and 0.1% formic acid. Another aliquot was also analyzed using acidic positive ion conditions, which was chromatographically optimized for more hydrophobic compounds. The extract was gradient eluted from the C18 column using methanol, acetonitrile, water, 0.05% PFPA and 0.01% formic acid and was operated at an overall higher organic content. Another aliquot was analyzed using basic negative ion optimized conditions using a separate dedicated C18 column. The basic extracts were gradient eluted from the column using methanol and water with 6.5 mM Ammonium Bicarbonate at pH 8. The fourth aliquot was analyzed via negative ionization following elution from a HILIC column (Waters UPLC BEH Amide 2.1 × 150 mm, 1.7 µm) using a gradient consisting of water and acetonitrile with 10 mM ammonium formate, pH 10.8. The MS analysis alternated between MS and data-dependent MS^n^ scans using dynamic exclusion. The scan range covered 70–1000 *m*/*z*.

### 2.5. Data Extraction, Compound Identification and Metabolite Quantification

Raw data were extracted, peak-identified and QC processed by Metabolon (Metabolon, Inc., Morrisville, NC, USA). Compounds were identified by comparison to library entries of purified standards or recurrent unknown entities. Biochemical identifications are based on three criteria: retention index within a narrow RI window of the proposed identification, accurate mass match to the library ±10 ppm, and the MS/MS forward and reverse scores between the experimental data and authentic standards. The MS/MS scores are based on a comparison of the ions present in the experimental spectrum to the ions present in the library spectrum. Peaks were quantified by Metabolon, Inc. using the area-under-the-curve with a data normalization step was performed to correct variation resulting from instrument inter-day tuning differences.

### 2.6. Statistical Analysis

Statistical analysis was performed by Metabolon as well as using MetaboAnalyst 5.0 (https://www.metaboanalyst.ca, accessed date on 6 June 2021) for pathway and enrichment analyses. Following log transformation and imputation of missing values, if any, with the minimum observed value for each compound, Welch’s two-sample *t*-test was used to identify biochemicals that differed significantly between experimental groups, while outliers were identified using the ROUT method. Biochemicals that achieved statistical significance (*p* ≤ 0.05) and demonstrated a low estimate of false discovery rate (*q* > 0.10) were included in this analysis.

## 3. Results

### 3.1. Clinical Characteristics of the Cohort

We identified mothers with infants born <37 weeks with a first-time radiologic or pathologic diagnosis of NEC. Among these mother-infant dyads, 18 were selected and age-matched non-NEC control infants and their mothers were also identified. No significant differences in the baseline characteristics were observed between these two groups (Table 1). We next identified mothers with non-NEC control infants born <37 weeks with stratification of infants into groups with ≤1.0 kg birth weight (*n* = 13) and >1.5 kg birth weight (*n* = 15), which were not age-matched. Infants in the ≤1.0 kg group had a median estimated gestational age (EGA) of 27 weeks at birth (interquartile range (IQR) 25, 28) and median birth weight of 805 g (IQR 595, 947.5) (Table 1). Infants in the >1.5 kg group had a median EGA of 33 weeks at birth (IQR 32, 34) and median birth weight of 2140 g (IQR 1583, 2335). Infants with a smaller birth weight of ≤1.0 kg were more likely to be small for gestational age (*p* = 0.03).

### 3.2. Multivariate Analysis of Milk Metabolic Profiles

**Figure 1:**
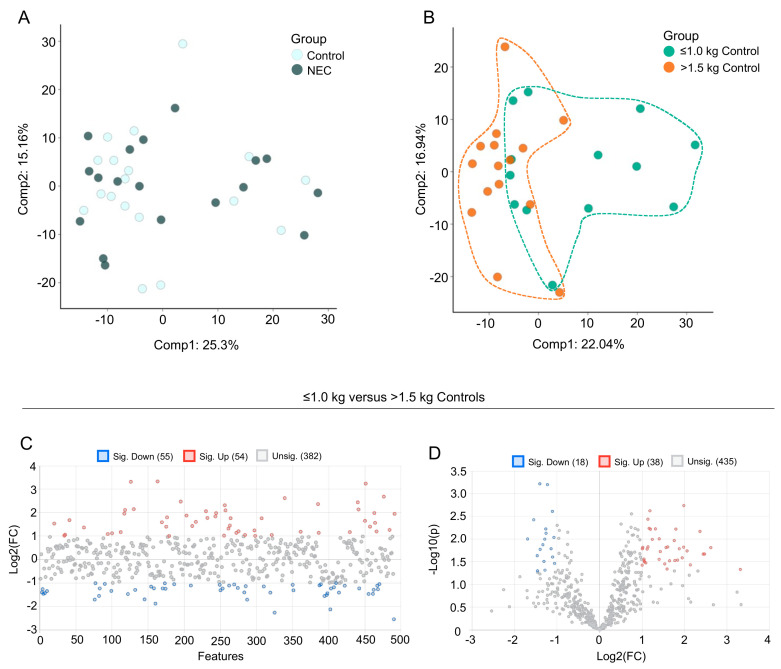
Principal component analysis (PCA) and fold change analysis of human milk samples. PCA of the data reveals (**A**) segregation of breast milk samples from mothers of control infants and infants with NEC and (**B**) segregation of breast milk samples from mothers of control infants with birth weight ≤1.0 kg compared with birth weight >1.5 kg. (**C**) Fold change analysis of the absolute value of change between the ≤1.0 kg versus >1.5 kg birth weight control groups using a fold change threshold of 2.0. (**D**) Volcano plot analysis of ≤1.0 kg versus >1.5 kg birth weight control groups using a fold change threshold of 2.0 and *p* < 0.05.

Data were analyzed by Metabolon, Inc. and further analyzed using MetaboAnalyst 5.0 for additional statistical and enrichment analysis. A Principal Component Analysis (PCA) was performed for the assessment of possible clusters or outliers among the NEC versus control (Figure 1A) and the ≤1.0 kg versus >1.5 kg birth weight control (Figure 1B) groups. A total of 631 named and 37 unnamed biochemicals were detected in the milk sample dataset. In total, only two significant differences in biochemicals detected in the NEC group when compared with the age-matched controls, indicating that the difference in metabolomic profiles between the NEC and control groups was minimal. There were 159 biochemicals with significant differential expression in the ≤1.0 kg versus >1.5 kg comparison groups, including 111 biochemicals with a *q-*value < 0.10 suggestive of a low risk of false discovery. As there were minimal significant differences in the milk metabolites from mothers with NEC infants compared with non-NEC controls, we next focused on the differences in milk metabolites between mothers with infants with a birth weight of ≤1.0 kg compared with >1.5 kg. A fold change analysis of the absolute value of change between the ≤1.0 kg versus >1.5 kg birth weight control groups using a fold change threshold 2.0 revealed 54 significantly increased and 55 significantly decreased concentrations of metabolites between the birth weight control groups (Figure 1C). Further, a volcano plot analysis of ≤1.0 kg versus >1.5 kg birth weight control groups using a fold change threshold of 2.0 and *p* < 0.05 revealed 38 significantly increased and 18 significantly decreased concentrations of detected metabolites (Figure 1D).

**Figure 2:**
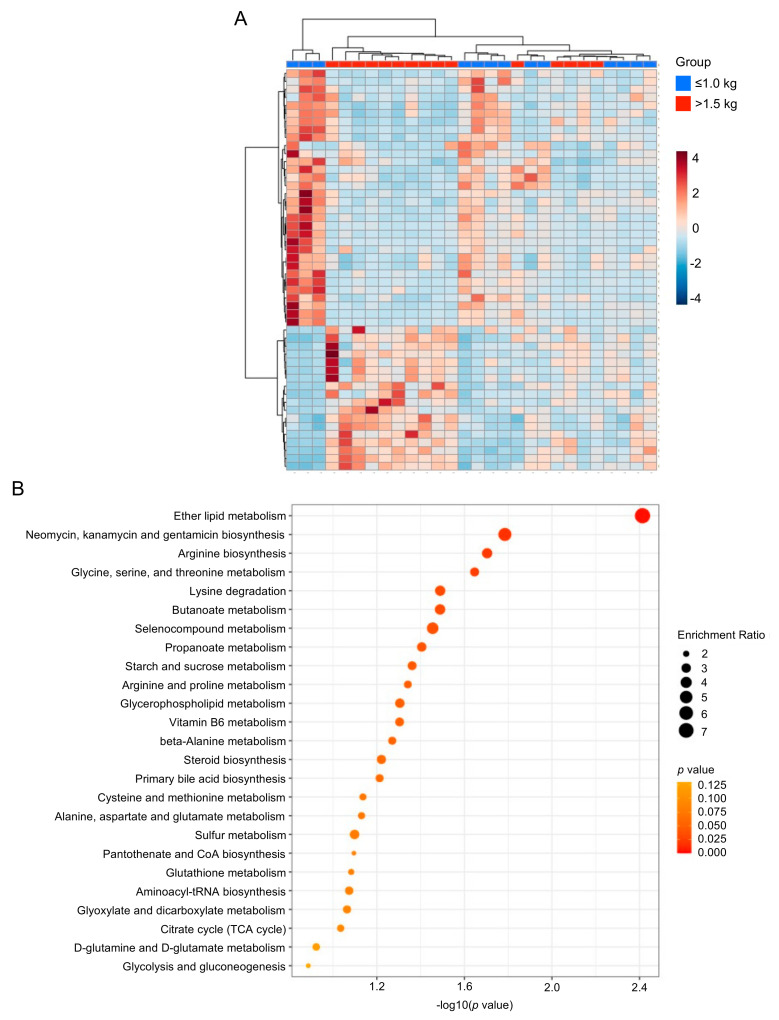
Heat map and enrichment analysis of control group human milk samples. (**A**) Hierarchical clustering heat map of the top 50 differentially expressed metabolites in the ≤1.0 kg (blue) versus >1.5 kg (red) birth weight control groups by T-test. (**B**) Quantitative enrichment analysis reveals the top 25 enriched metabolite sets in the milk of mothers with infants ≤1.0 kg birth weight compared with >1.5 kg birth weight.

We next performed exploratory statistical analyses to identify overall differences in metabolites. Hierarchical clustering heat map analysis (Ward’s method) of the top 50 differentially expressed metabolites in the ≤1.0 kg (blue) versus >1.5 kg (red) birth weight controls by T-test revealed distinct clusters of significantly increased or decreased metabolites (Figure 2A). Among the birth weight control groups, a metabolite set enrichment analysis (MSEA) reveals the top 25 enriched and biologically meaningful metabolites in the milk of mothers with infants ≤1.0 kg birth weight compared with >1.5 kg birth weight, including “ether lipid metabolism” (*p* = 0.0039); “neomycin, kanamycin and gentamicin biosynthesis” (*p* = 0.016); “arginine biosynthesis” (*p* = 0.02); “glycine, serine and threonine metabolism” (*p* = 0.023); “lysine degradation (*p* = 0.032); “butanoate metabolism” (*p* = 0.032), “selenocompound metabolism” (*p* = 0.035) and “propanoate metabolism” (*p* = 0.039) (Figure 2B).

### 3.3. Fatty Acid Composition of Maternal Milk Varies by Infant Birth Weight

**Figure 3:**
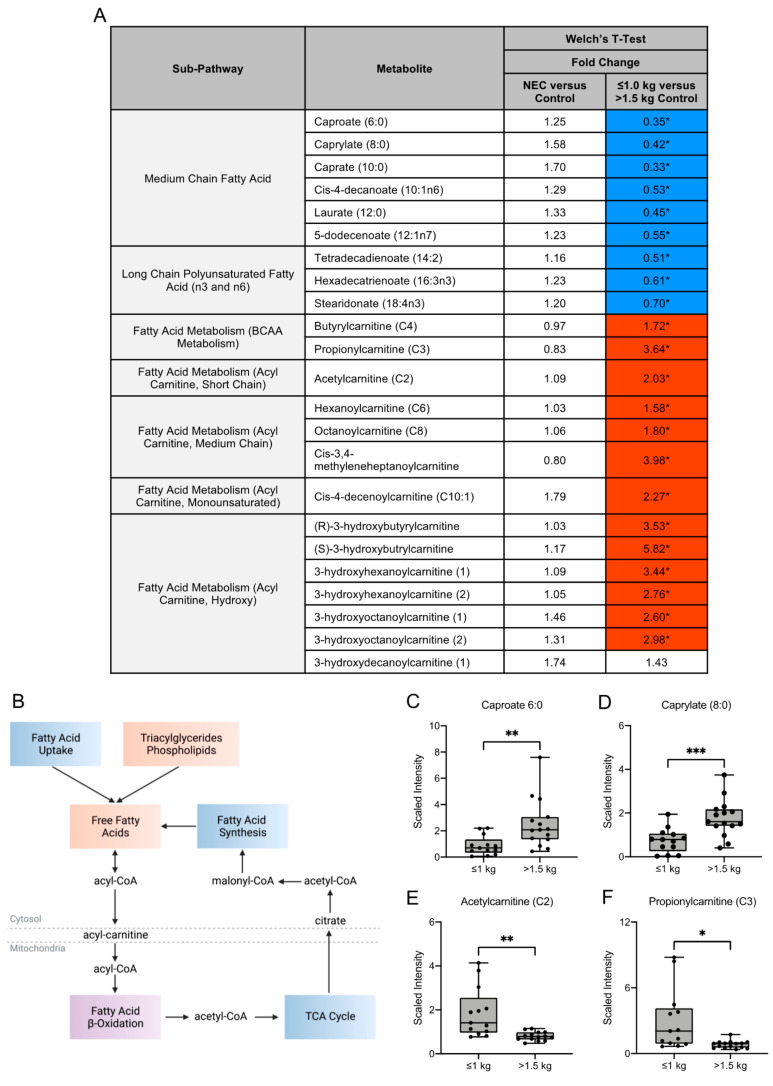
Metabolites in the fatty acid metabolism sub-pathways vary among birth weight control milk samples. (**A**) Fold change concentration of metabolites within fatty acid metabolism sub-pathways. Red cells indicate mean values that are significantly higher and blue cells indicate mean values that are significantly lower for the designated comparison. (**B**) Fatty acids destined for oxidation are conjugated to carnitine and acylcarnitines are transported into the mitochondria for fatty acid beta-oxidation. Figure created with BioRender.com. (**C**–**F**) Boxplots of concentration levels of caproate (6:0), caprylate (8:0), acetylcarnitine (C2) and propionylcarnitine (C3) between the birth weight control groups. * *p* ≤ 0.05, ** *p* < 0.01, *** *p* < 0.001 by Welch’s *T*-Test. TCA = tricarboxylic acid.

Maternal milk composition of fatty acids (FA) influences neonatal growth, development and immune function and can be impacted by the dietary intake of the mother. Several medium-chain FA and long-chain polyunsaturated FA (e.g., caproate (6:0), caprylate (8:0), caprate (10:0) and hexadecatrienoate (16:3n3)) were significantly lower in concentration in maternal milk in the ≤1.0 kg control group when compared to the >1.5 kg control group (Figure 3A,C,D). Long-chain fatty acids (LCFA) destined for oxidation are conjugated to carnitine and the acylcarnitines are then transported into the mitochondria for fatty acid beta-oxidation (Figure 3B). We observed several carnitine-conjugated fatty acid metabolites such as acetylcarnitine (C2) (Figure 3E) and propionylcarnitine (C3) (Figure 3F) to be significantly higher in the ≤1.0 kg control group when compared to the >1.5 kg control group. Overall, no significant differences in FA were identified between the NEC and control groups.

### 3.4. Human Milk Oligosaccharide, Amino Sugar and Amino Acid Components in Maternal Milk Differ by Infant Birth Weight

**Figure 4:**
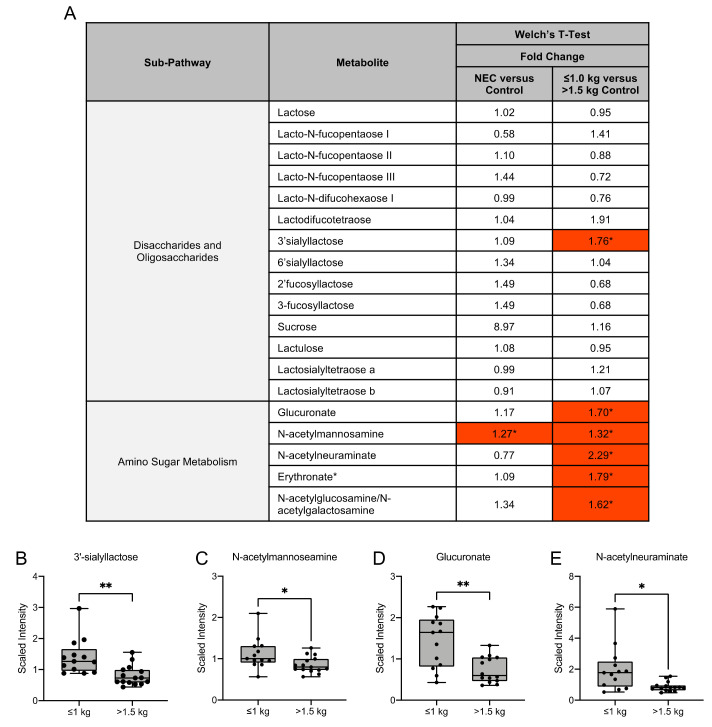
Human milk oligosaccharide and amino sugar composition of human milk differs among birth weight control milk samples. (**A**) Fold change concentration of human milk oligosaccharides and amino sugar metabolites in maternal milk. Red cells indicate mean values that are significantly higher and blue cells indicate mean values that are significantly lower for the designated comparison. (**B**–**E**) Boxplots of concentration levels of 3′sialyllactose, N-acetylmannosamine, glucuronate and N-acetylneuraminate between the birth weight control groups. * *p* ≤ 0.05, ** *p* < 0.01 by Welch’s *T*-Test.

Human milk oligosaccharides (HMOs) are complex sugars in breast milk with prebiotic properties and serve as metabolic substrates with targeted antimicrobial activity. We examined several HMOs and HMO-related compounds in this study (Figure 4A). Of these compounds, there were no significant differences in HMO concentrations in milk from mothers of infants with NEC and healthy controls (Figure 4A). Among the control groups, the HMO 3′sialyllactose (3′SL) was significantly increased in concentration in the milk of mothers with infants ≤1.0 kg birth weight when compared to infants of >1.5 kg birth weight (Figure 4B). No significant difference in lactose concentration was observed among any of the comparisons.

Amino sugars are components of bacterial cell walls and serve as a key by-product of HMO metabolism [18]. We identified that the amino sugar N-acetylmannosamine was significantly higher in the NEC group when compared to the control group, while it was significantly higher in the ≤1.0 kg control group when compared to the >1.5 kg control group (Figure 4C). The amino sugars glucuronate, N-acetylneuraminate and erythronate and the amino sugar isobar N-acetylglucosamine/N-acetylgalactosamine were significantly higher in the ≤1.0 kg control group when compared to the >1.5 kg control group (Figure 4A,D,E). 

**Figure 5:**
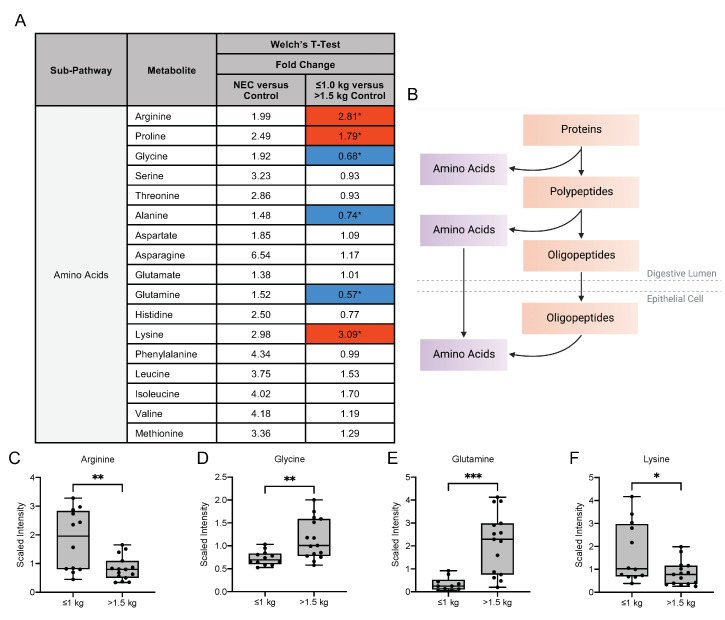
Amino acid composition of breast milk varies by birth weight in control infants. (**A**) Fold change concentration of amino acids in maternal milk. Red cells indicate mean values that are significantly higher and blue cells indicate mean values that are significantly lower for the designated comparison. (**B**) Intake of amino acids in breast milk is followed by catabolism by the intestinal mucosa for degradation to dietary amino acid precursors. Figure created with BioRender.com. (**C**–**F**) Boxplots of concentration levels of arginine, glycine, glutamine and lysine between the birth weight control groups. * *p* ≤ 0.05, ** *p* < 0.01, *** *p* < 0.001 by Welch’s *T*-Test.

As free amino acids account for 3–5% of the total amino acids in mother’s milk, we next sought to investigate the differences in amino acid metabolites in mother’s milk [19] (Figure 5A). Dietary ingestion of amino acids in breast milk results in intestinal mucosal amino acid catabolism by the small intestine, resulting in the conversion to dietary amino acids that serve as precursors for substances required for maintenance of intestinal integrity (Figure 5B) [20]. In our untargeted metabolomic analysis of amino acid metabolites, we observed no significant difference in the fold change expression of these metabolites in the milk of mothers with infants with NEC when compared to the milk of mothers with healthy control infants. In contrast, significant differences in several amino acid metabolites were detected among the control groups. Milk from mothers with infants of birth weight ≤1.0 kg had a significantly increased concentration of the amino acids arginine, proline and lysine when compared to the milk of mothers of infants with birth weight >1.5 kg (Figure 5C–F). Additionally, we observed a significantly decreased fold change concentration of amino acids glycine, alanine and glutamine in the ≤1.0 kg birth weight group relative to the >1.5 kg birth weight infants. The significant differences in these metabolites between birth weight control groups provide insight into the potential need for exogenous supplementation of amino acids that may provide growth and development in the newborn. 

### 3.5. Metabolic Differences in the Microbiome-Derived and Vitamin Metabolites in Maternal Milk

**Figure 6:**
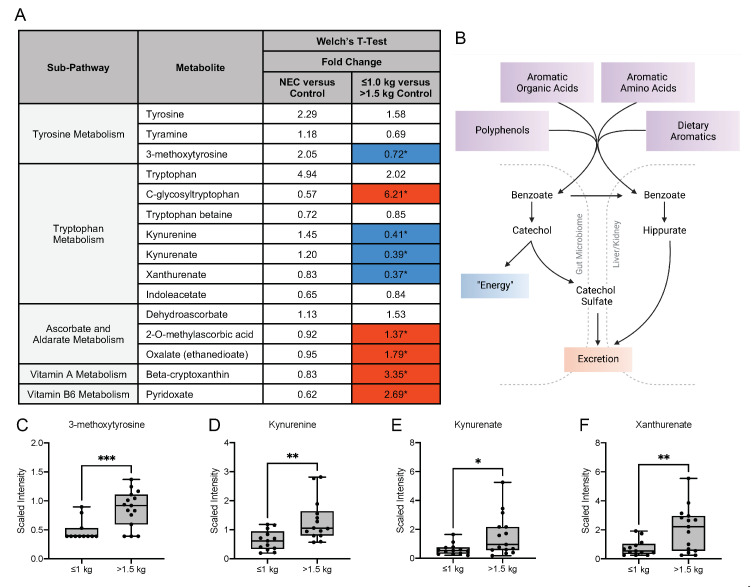
Tyrosine, tryptophan and vitamin metabolites in maternal milk vary among birth weight control groups. (**A**) Fold change concentration of tyrosine, tryptophan and vitamin metabolites in maternal milk. Red cells indicate mean values that are significantly higher and blue cells indicate mean values that are significantly lower for the designated comparison. (**B**) Microbial action within the intestine facilitates the metabolism of aromatic amino acids and vitamins. Figure created with BioRender.com. (**C**–**F**) Boxplots of concentration levels of 3-methoxytyrosine, kynurenine, kynurenate and xanthurenate between the birth weight control groups. * *p* ≤ 0.05, ** *p* < 0.01, *** *p* < 0.001 by Welch’s *T*-Test.

Interactions with the intestinal microbiome facilitate the metabolism of aromatic amino acids, such as phenylalanine, tyrosine and tryptophan and vitamins [21,22]. In our analysis, no significant differences in the fold change concentration of tyrosine, tryptophan or vitamin metabolites were detected in the milk from mothers of infants with NEC compared with healthy control infants (Figure 6A). Prior studies have implicated the importance of endogenous tryptophan metabolites in maintaining intestinal homeostasis related to the metabolic activity of the gut microbiota and mucosal immune reactivity (Figure 6B) [23]. Our untargeted analysis detected significant differences in fold change concentration in several of these metabolites among the control groups. The tyrosine metabolite 3-methoxytyrosine was significantly lower in the ≤1.0 kg control group compared with the >1.5 kg control group (Figure 6C). The tryptophan metabolite C-glycosyltryptophan, which has a known associated increase with chronological age in humans, was significantly increased in the ≤1.0 kg group [3]. However, the fold change concentration of several tryptophan metabolites was significantly decreased in the ≤1.0 kg group when compared to the >1.5 kg birth weight control, including kynurenine, kynurenate and xanthurenate (Figure 6D–F).

Interestingly, the fold change concentration of several vitamins was significantly increased in the ≤1.0 kg group when compared to the >1.5 kg birth weight group, including oxalate, beta-cryptoxanthin (a vitamin A metabolite) and pyridoxate (a vitamin B6 metabolite). There were no observed differences in vitamin metabolite levels in the NEC versus control comparison.

### 3.6. Differences in Oxidative Stress-Related, Glycolytic and Energy Metabolites

**Figure 7:**
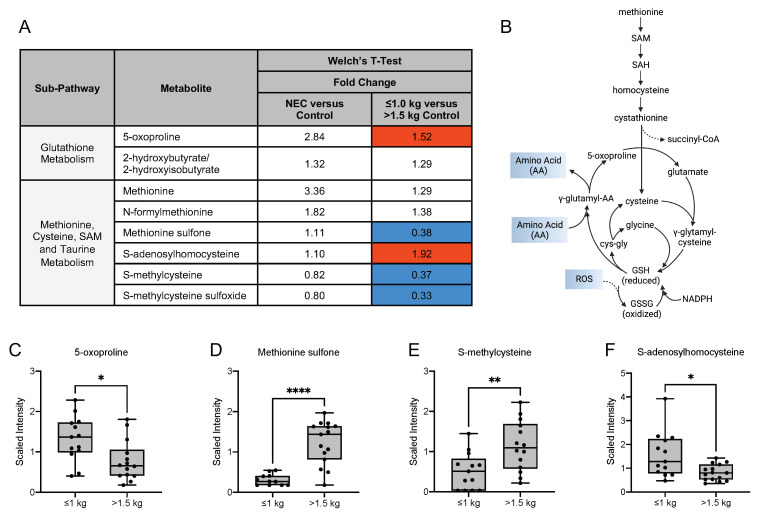
Compounds derived from glutathione, methionine, cysteine, S-adenosylmethionine and taurine metabolism differ among the birth weight control groups. (**A**) Fold change concentration of metabolites in maternal milk. Red cells indicate mean values that are significantly higher and blue cells indicate mean values that are significantly lower for the designated comparison. (**B**) Metabolism of methionine, an essential amino acid and a major target of reactive oxygen species, is a source for cysteine, which is required for glutathione synthesis. (**C**–**F**) Boxplots of concentration levels of 5-oxoproline, methionine sulfone, S-methylcysteine and S-adenosylhomocysteine between the birth weight control groups. * *p* ≤ 0.05, ** *p* < 0.01, **** *p* < 0.0001 by Welch’s *T*-Test. GSH = glutathione, GSSG = glutathione disulfide, NADPH = nicotinamide adenine dinucleotide phosphate, SAH = S-adenosylhomocysteine, SAM = S-adenosylmethionine.

Oxidative stress can occur in the setting of an imbalance between free radical or reactive oxygen species (ROS) and antioxidant defenses. Several markers of oxidative stress have been identified in human breast milk [24]. Therefore, we next investigated breast milk metabolites from sub-pathways related to glutathione metabolism and methionine, cysteine, S-adenosylmethionine (SAM) and taurine metabolism (Figure 7A). Methionine, an essential amino acid, is a source of cysteine, which is required for glutathione synthesis (Figure 7B) [25,26]. Cysteine is considered an essential amino acid in preterm neonates, due to the decreased ability to convert cystathionine to cysteine [25]. Analysis of oxidative stress-related metabolites derived from the glutathione pathway revealed no significant difference was observed between the NEC and control groups. A significantly increased concentration of 5-oxoproline was observed in the ≤1.0 kg control group when compared to the >1.5 kg control group (Figure 7C). Oxidative stress-related metabolites related to methionine, cysteine, SAM and taurine metabolism also demonstrated notably significant differences between the two control groups. Methionine sulfone (Figure 7D), S-methylcysteine (Figure 7E), and S-methylcysteine sulfoxide were decreased in the concentration of milk from mothers of infants with birth weight ≤1.0 kg when compared to the >1.5 kg birth weight control group. In contrast, S-adenylhomocysteine (Figure 7F) was significantly increased in the ≤1.0 kg control group when compared to the >1.5 kg birth weight control group. Additionally, within the glycolysis, gluconeogenesis and pyruvate metabolism sub-pathway, a significant decrease in the fold change concentration of glucose (0.72; *p* ≤ 0.05) was observed in the ≤1.0 kg control group when compared to the >1.5 kg control group with no difference between the NEC and control groups. No differences were identified in 3-phosphoglycerate, pyruvate or lactate among any of the studied comparisons. Further, within the TCA cycle sub-pathway, a significant increase in fold change concentration of succinylcarnitine (C4-DC) (2.62; *p* ≤ 0.05) was observed in the ≤1.0 kg control group when compared to the >1.5 kg group, with no difference identified between the NEC and control groups. These findings provide greater insight into the potential nutritional outcomes and disease risks in infants receiving milk with varying compositions of oxidative stress-related, glycolytic and energy metabolites.

## 4. Discussion

Human milk is the gold standard for nutritional support for growth and development during infancy, and the complex composition of human milk encompasses macronutrients, micronutrients, antimicrobial and bioactive factors [4,8,27]. The composition of human milk is dynamic and influenced by a variety of maternal and environmental factors [4,8,27]. These antimicrobial and bioactive factors have multipurpose properties, including established protective roles against the development of neonatal disorders such as NEC. Maternal milk metabolomics is a growing field of research seeking to understand the complexities of human milk, its relationship to maternal health and diet and the overall impact on infant health. In this study, we explored the metabolome of human milk samples collected from mothers of infants that developed NEC and healthy controls and further characterized the milk metabolome of mothers with control neonates with birth weight of ≤1.0 kg compared with birth weight >1.5 kg. Though this untargeted metabolomics analysis identified minimal significances between milk from mothers of infants with NEC and non-NEC healthy controls, significant variations were observed in milk from mothers of infants among the two control birth weight groups.

Key nutrients in human milk, such as lipids and fatty acids, can be influenced by the health and diet of the mother and can impact neonatal growth and intestinal development [28]. The relationship between maternal diet and adiposity and nutritional composition of breast milk demonstrates the impact of dietary and environmental influences on the nutritional content of maternal milk [29,30]. Prior studies have shown that polyunsaturated fatty acids, linoleic acid and total omega-6 fatty acids in breast milk were lower in concentration in older mothers [31]. It has been further identified that monounsaturated fatty acid content in human milk is lower and the omega-6:omega-3 polyunsaturated fatty acid ratio and leptin concentrations are higher in mothers with a higher body mass index (BMI) [31,32]. Importantly, the complex role of enteral long-chain polyunsaturated fatty acid (LCPUFA) on intestinal inflammation and the incidence of NEC and death in a rat model has been previously described [33,34]. In our analysis, we observed a significant decrease in long-chain polyunsaturated fatty acids including tetradecadienoate (14:2), hexadecatrienoate (16:3n3) and stearidonate (18:4n3) in the milk of mothers of ≤1.0 kg control infants, suggesting a possible increased risk of intestinal inflammation and NEC in this group. Additionally, the neonatal gut microbiome can be influenced by exposure to dietary saturated and monounsaturated fatty acids [28]. Specifically, breast milk with higher concentrations of monounsaturated fatty acids (MUFAs) is associated with a milk microbiota with decreased *Lactobacillus* and *Bifidobacterium*, which are necessary for maintaining intestinal homeostasis and barrier integrity [35,36]. In our untargeted metabolomic analysis, cis-4-decenoylcarnitine (C10:1), a monounsaturated acylcarnitine, was significantly increased in the milk of mothers of infants in the >1.5 kg control group, implicating a potential decrease in beneficial gut microbiota in the ≤1.0 kg control group, further placing these infants at risk of dysbiosis and risk of NEC.

Human milk contains a high amount of HMOs and other complex oligosaccharides that can be used by intestinal microbial species as carbon and energy sources [8,18,37,38,39]. The prebiotic and immunomodulatory functions of HMOs can influence the composition of the microbiome and intestinal maturation [18,37,38,39]. While over 200 HMOs have been identified, it is known that not every mother synthesizes the same set of oligosaccharides and the HMO composition is highly influenced by the presence of the *FUT2* and *FUT3* secretor genes [11,40]. Prior studies of maternal milk have identified a higher concentration of HMOs in postpartum time-matched milk from mothers of full-term infants [41]. Additionally, HMOs have a suggested role in intestinal maturation in an in vitro epithelial model in which treatment with HMOs induced epithelial cell differentiation maturation [42]. Several preclinical animal studies as well as human studies support the importance of HMOs in reducing the risk of developing NEC [37,43,44,45]. Interestingly, certain *Bifidobacterium* species are known to consume HMOs such as N-acetyl-D-glucosamine, suggesting that the complex and dynamic gut bacterial responses to HMOs may influence the infant gut microbiome composition [38]. The increased concentration of N-acetylglucosamine observed in our analysis in the milk of mothers with lower birth weight control infants may suggest a protective role of maternal milk in developing the infant microbiome and protecting against the dysbiosis observed in NEC. Amino sugars are biosynthesized by primary and secondary metabolism routes by the conversion of a monosaccharide to an amino sugar derivative [46]. Amino sugar components of bacteria have been well established. For example, the amino sugar N-acetyl-L-fucosamine is present in lipopolysaccharides of some bacteria such as *Pseudomonas aeruginosa*. The amino sugar kanosamine is produced by *Bacillus* and *Streptomyces* species and exhibits antimicrobial action [46]. The observed differences in HMOs and amino sugars in our data can contribute to identifying how customized nutritional interventions can be used to meet the needs or dietary deficiencies of the newborn. These findings suggest potential impacts of alterations in microbial colonization of the infant and the risk of developing diseases associated with dysbiosis such as NEC.

A higher risk of developing NEC is also associated with a neonatal deficiency in circulating amino acids, including glutamine and arginine [47]. Therefore, the amino acid composition of human milk has been an important area of investigation. Prior studies of neonatal rat models with experimental NEC receiving exogenous treatment of glutamine demonstrated reduced intestinal pathology injury scores and decreased mRNA expression of innate immune receptors toll-like receptor (TLR)-2 and TLR-4 [48]. Further, a prior analysis of milk from mothers of term neonates revealed that glutamine and glutamic acid were amongst the most abundant free amino acids during the first three months of lactation [19]. Notably, our analysis identified a significantly decreased concentration of glutamine in the milk of mothers with infants ≤1.0 kg birth weight, suggesting an increased predisposition to the development of NEC in this weight group.

Amino acids such as tryptophan and its endogenous metabolites have established roles in intestinal immunity and homeostasis and prior investigations have shown that milk from mothers of preterm infants contains less free tryptophan levels than milk from mothers of term infants [5]. Differences in the aromatic amino acids tyrosine or tryptophan metabolites may alter infant microbial composition and activity, affecting breast milk nutritional content and contributing to NEC development. Anaerobic fermentation of tyrosine and tryptophan by intestinal bacteria results in the derivation of phenols as well as indoles, which have recently shown benefit in attenuating intestinal inflammation in NEC [6,7]. The metabolism of tryptophan by intestinal bacteria along the kynurenine pathway results in metabolites that are implicated in microbial defense, immunoregulation and antioxidant activity in the intestine [23,49,50]. Indoleamine 2,3-dioxygenase 1 (IDO1) is an enzyme that causes tryptophan degradation to produce the breakdown product L-kynurenine, which activates aryl hydrocarbon receptor signaling, and in the gastrointestinal tract, promotes local interleukin (IL)-22 production by innate lymphoid cells [23]. In this analysis, we identified several tryptophan metabolites decreased in concentration in maternal milk in the lower birth weight control group, including kynurenine, kynurenate and xanthurenate. Importantly, prior studies have shown that a dietary lack of tryptophan impairs intestinal immunity in mice and alters the gut microbiome, indicating a role for the activation of the AhR-IL-22 axis in mucosal immune homeostasis of the gut [23]. We have recently shown that treatment with IL-22 attenuates intestinal inflammation in experimental murine NEC by promoting epithelial regeneration [51]. Additional investigations have shown that indole-3-lactic acid, a metabolite of breast milk tryptophan secreted by *Bifidobacterium longum* subspecies *infantis* attenuates the inflammatory response in vitro in human immature intestinal organoids stimulated with human IL-1B [50] and in LPS-treated adult intestinal epithelial cells [49]. Therefore, characterizing the concentration of tryptophan in maternal milk and identifying preterm and low birth weight infants at risk of a relative nutritional deficiency may identify opportunities for tailored nutrition for infants at risk of the pathogenesis of intestinal diseases such as NEC.

Additionally, the diagnosis of oncologic and metabolic childhood disorders is associated with the concentrations of serum tyrosine metabolites. The compound 3-methoxytyramine, the O-methylated metabolite of dopamine, is useful for the diagnosis of neuroblastoma or dopamine-producing pheochromocytomas and paragangliomas [52] and elevated serum levels are associated with aromatic L-amino-acid decarboxylase deficiency [53]. Although no difference in 3-methoxytyramine levels was observed in the maternal milk in the NEC and control groups, the decreased levels in milk of mothers of infants of ≤1.0 kg birth weight may suggest an overall relative dietary deficiency in this preterm infant weight group, the implications of which require further investigation.

Preterm infants also rely on the metabolic profile of breast milk as a source for methionine, an essential amino acid [25]. Methionine is involved in the defense against oxidative stress and has a role in the formation of cysteine from homocysteine via cystathionine γ-lyase, an enzyme which is absent in the fetal liver and has reduced level of activity in the newborn period [25,26]. As a deficiency in methionine can impact growth, it is important to investigate breast milk methionine metabolites to evaluate the impacts of dietary deficiencies [26]. Our data revealed a decreased concentration of methionine sulfone, S-methylcysteine and S-methylcysteine sulfoxide in the ≤1.0 kg control group, which suggests a relative dietary deficiency, the recognition of which provides an opportunity for optimization of neonatal nutritional intake. Additionally, the glutathione system plays an important role in the protection against oxidative stress through the scavenging of reactive oxygen species [54]. Therefore, defining the relationship between maternal oxidative stress and neonatal oxidative stress is essential in advancing our understanding of the impact of the perinatal period on neonatal outcomes. As our data demonstrated an increased concentration of 5-oxoproline in the milk of mothers with infants ≤1.0 kg birth weight compared to >1.5 kg, this suggests an altered state of oxidative stress within the breast milk of the lower birth weight group. The capacity of oxidative stress metabolites in maternal milk to impact inflammation and oxidative stress in the receiving infant requires further investigation.

Fat-soluble and water-soluble vitamins are among the nutrients in human milk most strongly affected by the dietary intake of the breastfeeding mother [30,55]. Therefore, understanding the variability of vitamin content in maternal milk can guide tailored nutrition of the preterm infant at risk of dietary deficiency and can influence milk fortification strategies. It is well known that the transfer of vitamin D and its metabolites from plasma to milk is limited. Additionally, human breast milk contains low concentrations of vitamin K and there is strong evidence for an increased incidence of late hemorrhagic disease of the newborn in those who are breastfed [56]. Kamao and colleagues used LC/MS to quantify concentrations of fat-soluble vitamins in human breast milk from 82 mothers and identified that concentrations of 25(OH)D in breast milk were significantly lower when compared to the 25(OH)D concentration in plasma [56]. Redeuil and colleagues compared human milk at equivalent post-partum ages and assessed for concentrations of vitamins. When compared at equivalent post-menstrual age, preterm maternal milk was significantly higher for vitamins B1, B2, B3, B6 and B9 and also had lower concentrations of vitamins A, E, beta-carotene, beta-cryptoxanthin and lutein when compared to term milk [57]. Similarly, in our analysis, we identified a significantly increased concentration of pyridoxate, a vitamin B6 metabolite, and beta-cryptoxanthin, a carotenoid, in the milk of mothers with preterm control infants with a birth weight of ≤1.0 kg.

Overall, these data reveal a comprehensive metabolic fingerprint of maternal milk for infants of ≤1.0 kg birth weight compared to >1.5 kg birth weight with minimal overall differences between milk from mothers of infants with NEC and healthy controls. Although the overall significance of these data require further investigation into the mechanism and consequences of these differences, the untargeted metabolomic analysis provides an opportunity for exploration of potential diagnostic or treatment targets in high-risk preterm infants.

## 5. Conclusions

Human milk metabolomics is a developing field for the exploration of the relationship between the metabolic composition of breast milk, maternal phenotypes, and infant health. While the complexities of maternal milk require ongoing investigation, new technologies and new analytical methodologies are essential in the future of research on the role of the comprehensive metabolome on the health of the infant. Though minimal significant metabolic differences were identified in the milk of mothers of infants with NEC and non-NEC controls, we identified significant differences in milk composition by infant birth weight. Understanding the complexities of human milk therefore provides important insight for the future of tailored nutrition of the newborn.

## Figures and Tables

**Table 1:** Cohort Characteristics.

	NEC	Control	*p* Value	≤1.0 kg	>1.5 kg	*p* Value
	*n* = 18	*n* = 18		*n* = 13	*n* = 15	
Male sex	11 (48%)	12 (52%)	0.58 ^a^	6 (46%)	12 (80%)	0.06 ^a^
Cesarean section delivery	13 (72%)	11 (61%)	0.48 ^a^	10 (77%)	8 (53%)	0.19 ^a^
Histologic chorioamnionitis	2 (11%)	2 (11%)	>0.99 ^a^	0 (0%)	1 (7%)	0.34 ^a^
Race			0.29 ^a^			0.03 ^a^
White	16 (89%)	12 (75%)		7 (54%)	13 (89%)	
Black/African American/Other	2 (11%)	4 (25%)		6 (46%)	2 (11%)	
Gestational Age (weeks)	28 (25, 33)	28 (26, 33)	0.94 ^b^	27 (25, 28)	33 (32, 34)	<0.0001 ^a^
Birth Weight (grams)	1020 (705, 1948)	1560 (840, 2015)	0.45 ^b^	805 (595, 947.5)	2140 (1583, 2335)	<0.0001 ^b^
Small for gestational age	2 (10%)	3 (12%)	0.79 ^a^	3 (19%)	0 (0%)	0.03 ^a^
Maternal Age (years)	29 (24, 33)	29 (27, 31)	0.88 ^b^	29 (23, 34)	29 (28, 33)	0.69 ^b^
Maternal Gravida	2 (1, 4)	2 (1, 4)	0.64 ^b^	2 (1, 2)	2 (1, 4)	0.45 ^b^
Maternal Betamethasone	14 (82%)	14 (82%)	>0.99 ^a^	11 (92%)	12 (80%)	0.40 ^a^
DOL of maternal milk collection	15 (7, 24)	15 (7, 23)	0.63 ^b^	9 (5, 21)	14 (13, 20)	0.24 ^b^
DOL of NEC development	17 (9, 21)	--	--	--	--	

Data are median (IQR) or *n* (%). DOL = day-of-life, NEC = necrotizing enterocolitis. ^a^ Chi-squared test, ^b^ Mann–Whitney U-test.

## Data Availability

The data presented in this study are available on request from the corresponding author.
